# The Role of Scar Bands in the Treatment of Fractures of the Maxillaries with Extensive Losses of Substance

**Published:** 1919

**Authors:** 


**The Role of Scar Bands in the Treatment of Fractures of the Maxillaries with Extensive Losses of Substance.** By MM. Gernez and Lemiere. *La Restauration Maxillo-faciale*, February, 1918.

The authors advocate a new therapeutical technic guiding the process of scar formation, keeping systematically certain parts of the scar tissue (deep layers and most of all intermaxillary bands) and removing all superficial layers.

They consider that all scars are composed of : a) a deep or mucous layer ; b) a superficial or cutaneous layer.

The deep layer is composed of : (1) Interfragmentary bands ; (2) Intermandibulo-tegumentary bands ; (3) Intermaxillary bands.

Following treatment is resorted to :

a) *Deep Layer.* (1) Interfragmentary bands are retained as long as they do not interfere with the adaptation of apparatus. They prevent anterior fragments from projecting forward.

(2) Intermandibulo-tegumentary bands should usually be removed.

(3) Intermaxillary bands should be kept, and their formation favored as much as possible. Continuous occlusion, if possible, favors greatly the formation of such bands of adequate length. Such bands acting concomitantly with prosthetic appliances greatly help in the restoration of the masticatory apparatus.

b) *Superficial Layer.* Cutaneous scars should be entirely resected.

The following case illustrates the author's report. The patient suffered from several wounds:

(1) Loss of substance of both the ascending and horizontal branches.

(2) Perforation in the palate's posterior third.

(3) Loss of the left edge and the anterior half of the tongue.

(4) Great loss of substance in the central part of the left cheek.

(5) Submaxillary fistula.

During the first 6 months splinters were removed and the process of cicatrization developed normally. The growth of both deep and superficial bands was favored as much as possible. Intermaxillary bands were pulled by means of weights, and the anterior fragment was controlled by intermaxillary forces.

After 6 months had elapsed the superficial scar tissue was resected, the edges of the operatory wound were dissected, and autoplasty by sliding process resorted to. Three months later still, control was realized by means of an apparatus acting concomitantly with the intermaxillary band. Mastication was henceforth possible, and one year after receipt of his wound the patient's appearance was very nearly normal again.

